# Identification of *Rhopalosiphum Padi* Virus 5′ Untranslated Region Sequences Required for Cryptic Promoter Activity and Internal Ribosome Entry

**DOI:** 10.3390/ijms160716053

**Published:** 2015-07-15

**Authors:** Ming-Kun Liu, Jie-Zue Lin, Tzyy-Rong Jinn, Hong-Lin Chan, Tzong-Yuan Wu

**Affiliations:** 1 Institute of Bioinformatics and Structural Biology, National Tsing Hua University, Hsinchu 300, Taiwan; E-Mails: lmk33@hotmail.com (M.-K.L.); blackeye514@hotmail.com (J.-Z.L.); 2 School of Chinese Medicine, China Medical University, Taichung 40402, Taiwan; E-Mail: jinn@mail.cmu.edu.tw; 3 Department of Bioscience Technology, Chung Yuan Christian University, Chungli 320, Taiwan

**Keywords:** baculovirus, cryptic promoter, dicistroviruses, IRES, RhPV

## Abstract

The 579-nucleotide 5′ untranslated region (5′UTR) of the *Rhopalosiphum padi* virus (RhPV) possesses a cross-kingdom internal ribosome entry site (IRES) activity that functions in insect, mammalian, and plant-derived *in vitro* translation systems, and six TAAG motifs within the DNA fragment encoding the RhPV 5′UTR were previously found to confer the RhPV 5′UTR with late promoter activity in baculovirus. In the present study, various truncated RhPV 5′UTR sequences were produced, and among them, a fragment of 110 bp ranging from nucleotides 309 to 418 was identified to be the shortest fragment responsible for the late promoter activity in baculovirus infected Sf21 cells. This 110 bp fragment contains a TAAG tandem repeat that retains more than 60% of the late promoter activity of the full length RhPV 5′UTR sequence. Further, IRES activity remained unchanged in all truncated RhPV 5′UTR constructs. Taken together, this novel 110 bp fragment having late promoter activity in baculovirus as well as IRES activity in mammalian cell, renders it a useful tool for the development of a “shuttle” bi-cistronic baculovirus gene expression and/or delivery vector.

## 1. Introduction

Translation in eukaryotes may be achieved in a cap-dependent or a cap-independent manner. An unusual cap-independent translation initiation mechanism is mediated by an RNA element termed an internal ribosomal entry site (IRES). Cap structure is an m^7^GpppN modification at the 5′ end of eukaryotic cellular mRNAs that can be recognized by the cap-binding complex (eukaryotic initiation factor complex 4F, eIF4F). The eIF4F protein complex can bind to the mRNA’s 5′ end cap structure and then the 40S small ribosomal subunit and associated initiation factors (termed the 43S pre-initiation complex) associate with the mRNA. The 43S pre-initiation complex then scans along the 5′ untranslated region (5′UTR) of the mRNA until an initiation codon is reached [1]. In contrast, IRES elements are long, highly structured regions within 5′UTRs. These elements were first discovered in *Picornaviridae* RNA genomes that lack the cap structure [2,3]. In these viruses, translation is driven by the complex RNA secondary structures in the IRES, which confer cap-independent translation. The IRES elements are not restricted to picornaviruses, but are also found in some retroviruses, flavivirus, and DNA viruses, e.g., HIV, HCV, and herpes simplex viruses were reported to contain IRES elements in their genomes [4]. Furthermore, IRES elements have also been found in the 5′UTRs of several cellular mRNAs [5]. Importantly, IRES-dependent translation has been reported to occur for a subset of cellular mRNAs when cap-dependent translation is impaired (e.g., under conditions of apoptosis, heat shock, viral infection, and at the G2/M phase of the cell cycle) [6,7,8].

Recently, many unusual IRES elements have also been found in an invertebrate viral family, *Dicistroviridae*. The family *Dicistroviridae* (genus *Cripavirus*) is also known as cricket paralysis virus-like viruses or picorna-like viruses [9]. The viruses in this family share physicochemical properties with members of the *Picornaviridae* [10]. The genomes of these viruses are small, single-stranded, positive-sense RNA molecules (approximately 9–10 kb) with two open reading frames (ORFs) that encode two polyproteins separated by an intergenic region (IGR). Dicistroviruses infect a range of insect hosts, from aphids and fire ants to *Drosophila* cells [11]. It has been shown that the 5′UTRs as well as the IGRs of these dicistroviruses contain IRES elements [12,13,14,15]. A recently described dicistrovirus IRES element is present in the 579-nucleotide (nt) 5′UTR of *Rhopalosiphum padi* virus (RhPV) named RhPV 5′UTR IRES. RhPV 5′UTR IRES possesses cross-kingdom IRES activity that functions efficiently in mammalian-, plant-, and insect-derived *in vitro* translation systems [15,16,17], although RhPV infection is restricted to aphid species [18]. Due to its cross-kingdom activity, the RhPV 5′UTR IRES has the potential to be used in eukaryotic cell expression vectors. Interesting, we have shown that the cDNA of the RhPV 5′UTR IRES possesses six TAAG motifs and can act as a cryptic promoter activity in baculovirus-infected Sf21 cells [19]. In this report, we demonstrate that a small fragment in the RhPV 5′UTR IRES cDNA, corresponding to nt 309–418, not only retains significant IRES activity in CHO cells, but also possesses baculovirus-dependent promoter activity in Sf21 insect cells. This novel transcription and translation coupled activity may be attributed to (1) tandem repeat TAAG motifs that can act as a baculovirus late or very late promoter transcription initiation and (2) the low level of secondary structure within this region, with only 40%–60% nucleotides being base-paired [16], which may permit access to ribosomes and protein factors for internal translation initiation.

## 2. Results and Discussion

### 2.1. Tandem Repeated TAAG Motifs Are Responsible for the Promoter Activity of RhPV IRES in Baculovirus Infected Sf21 Cells

**Figure 1:**
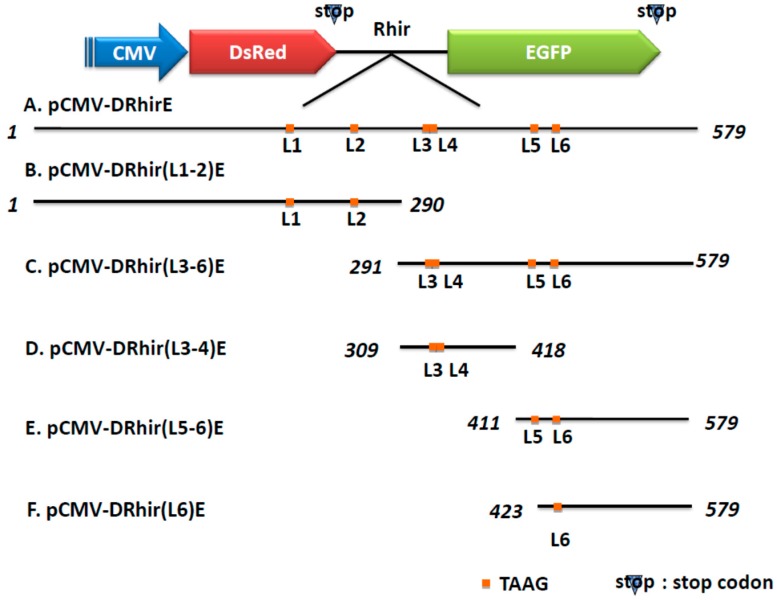
Schematic presentation of the truncated version of the RhPV 5′ IRES and bicistronic constructs. The indicated cDNA fragments were generated by PCR and inserted between the *DsRed* and *EGFP* genes in the bicistronic vector (**A**) pCMV-DRhirE which containing the completed 579 bps of RhPV IRES cDNA fragment and the six TAAG motifs are denoted with L1-6, respectively; (**B**) pCMV-DRhir(L1-2)E which containing the 1–290 bps of RhPV IRES cDNA fragment with the L1 and L2 TAAG motifs; (**C**) pCMV-DRhir(L3-6)E which containing the 291–579 bps of RhPV IRES cDNA fragment with the L3-L6 TAAG motifs; (**D**) pCMV-DRhir(L3-4)E which containing the 309–418 bps of RhPV IRES cDNA fragment with the L3 and L4 TAAG motifs; (**E**) pCMV-DRhir(L5-6)E which containing the 411–579 bps of RhPV IRES cDNA fragment with the L5 and L6 TAAG motifs; (**F**) pCMV-DRhir(L6)E which containing the 423–579 bps of RhPV IRES cDNA fragment with only the L6 TAAG motifs.

TAAG sequences are relatively rare in the AcMNPV genome and are found primarily in late or very late promoter regions [20]. However, the cDNA of the RhPV 5′UTR IRES possesses six TAAG motifs and can act as a cryptic promoter in baculovirus-infected Sf21 cells [19]. In order to characterize which of the six TAAG motifs mediated the promoter activity, truncated RhPV 5′UTR sequences were produced by making stepwise deletions from either ends; then, these truncated sequences were subject to functional promoter assays. For the sake of brevity, the six TAAG motifs in the RhPV 5′UTR IRES DNA sequence are designated as L1, L2, L3, L4, L5, and L6, respectively (Figure 1A). cDNA of the truncated RhPV 5′UTR as depicted in Figure 1A were respectively amplified by PCR using specific primers (Table 1) and the pCMV-DRhirE plasmid as template (Figure 1). These cDNA containing different TAAG motifs were then respectively inserted between two fluorescence reporter genes, *DsRed* and *EGFP*, in a baculovirus transfer vector (Figure 1). Plasmids thus produced were named pCMV-DRhir(L1-2)E, pCMV-DRhir(L3-6)E, pCMV-DRhir(L3-4)E, pCMV-DRhir(L5-6)E and pCMV-DRhir(L6)E (Figure 1B), respectively. These transfer vectors were co-transfected with viral DNA into the Sf21 cells and the resulting viruses were named vCMV-DRhir(L1-2)E, vCMV-DRhir(L3-6)E, vCMV-DRhir(L3-4)E, vCMV-DRhir(L5-6)E and vCMV-DRhir(L6)E, respectively.

**Table 1:** Primer sequences used to generate the truncated RhPV IRES.

Primer Name	Sequence	Nucleotide * Position
RH001-290F	GGC***GAATTC***GATAAAAGAACCTATAATC	1–19
RH001-290R	GGCCGG***GGATCC***GAATAAAATATAATAAAATAG	270–290
RH291-579F	GGA***GAATTC***ACCCCCCACATTAATCCC	291–308
RH291-579R	GGC***GGATCC***CGGGTATAAATAGATAAAG	564–579
RH309-418F	GGCCGG***GAATTC***AGTTAAAGCTTTATAAC	309–325
RH309-418R	GGC***GGATCC***ACTAAAAATTGTGAAAAATA	399–418
RH411-579F	GGCCGG***GAATTC***AATTTTTAGTTAAGATTTTAGC	411–432
RH291-579R	GGC***GGATCC***CGGGTATAAATAGATAAAG	564–579
RH423-579F	GGC***GAATTC***ATTTTAGCTTGCCTTAAG	423–440
RH291-579R	GGC***GGATCC***CGGGTATAAATAGATAAAG	564–579

GAATTC: EcoRI site; GGATCC: BamHI site 3. Experimental Section; * The numbers indicate the nucleotide position in the 5′UTR RhPV IRES.

**Figure 2:**
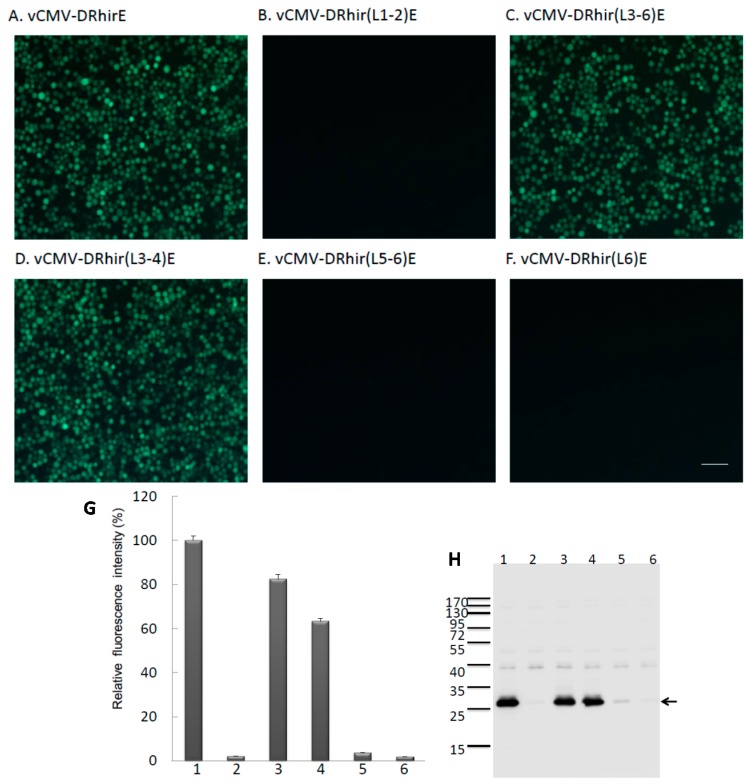
Identification of the TAAG motifs in the cDNA of RhPV 5′ IRES that are required for promoter activity in baculovirus infected Sf21 cells. Fluorescence micrographs of Sf21 cells infected with (**A**) vAcCMV-DRhirE (positive control); (**B**) vCMV-DRhir(L1-2)E; (**C**) vCMV-DRhir(L3-6)E; (**D**) vCMV-DRhir(L3-4)E; (**E**) vCMV-DRhir(L5-6)E or (**F**) vCMV-DRhir(L6)E at a multiplicity of infection of 1 at three days post-infection. Pictures were taken under a FITC channel with a 450/490-nm filter set using an exposure time of 900 ms; scale bar is 50 μm; (**G**,**H**) Quantitative analysis of promoter activity in the different TAAG motifs truncated cDNA fragments of RhPV 5′IRES by spectrofluorometer and Western blot; (**G**) Spectrofluorometric measurement of EGFP fluorescence in lysates (100 μL; 50 μg of protein) from Sf21 cells infected with (1) vAcCMV-DRhirE; (2) vCMV-DRhir(L1-2)E; (3) vCMV-DRhir(L3-6)E; (4) vCMV-DRhir(L3-4)E; (5) vCMV-DRhir(L5-6)E or (6) vAcCMV-DRhir423E. EGFP fluorescence emission was excited at 488 nm and monitored at 507 nm. The fluorescence intensity of EGFP was normalized to that of vAcCMV-DRhirE infected Sf21 cell lysates (100%); (**H**) Western blot analysis of cellular lysates from Sf21 cells infected with vAcCMV-DRhirE (Lane 1), vCMV-DRhir(L1-2)E (Lane 2), vCMV-DRhir(L3-6)E (Lane 3), vCMV-DRhir(L3-4)E (Lane 4), vCMV-DRhir(L5-6)E (Lane 5) or vCMV-DRhir(L6)E (Lane 6). Proteins (25 μg per lane) were separated by 12% SDS–PAGE and then electrotransferred onto PVDF membranes, which were then incubated with rabbit anti-GFP polyclonal antibody followed by an HRP-conjugated goat anti-rabbit secondary antibody. Detected protein bands were visualized on X-ray film using a Western blot chemiluminescence kit. Molecular mass markers (kDa) are indicated at left and the arrow indicated the EGFP protein band.

Sf21 cells infected with vCMV-DRhir(L1-2)E, which contains the L1 and L2 TAAG motifs, did not produce green fluorescence (Figure 2B). In contrast, Sf21 cells infected with vCMV-DRhir(L3-6)E, which contains the L3, L4, L5, and L6 TAAG motifs, produced green fluorescence, as did Sf21 cells infected with vAcCMV-DRhirE (Figure 2A,C). These results implied that the L3-L6, but not L1-L2 TAAG motifs, were responsible for the cryptic promoter activity of the RhPV 5′UTR IRES cDNA in baculovirus-infected Sf21 cells. The importance of the L3-L6 TAAG motifs to the cryptic promoter activity of the RhPV 5′UTR IRES cDNA was further validated by examining the respective fluoresce patterns of vCMV-DRhir(L3-4)E (containing L3,L4), vCMV-DRhir(L5-6)E (containing L5, L6) and vCMV-DRhir(L6)E (containing L6) infected Sf21 cells. It was found that only the vCMV-DRhir(L3-4)E infected Sf21 cells were capable of expressing EGFP proteins (Figure 2D); whereas both vCMV-DRhir(L5-6)E and vCMV-DRhir(L6)E infected Sf21 cells failed to emit green fluorescence (Figure 2E,F). However, we did not find evidence that some flanking sequences may be required for the promoter activity encoded in L1, L2, L5 or L6 but, rather that sequences had been deleted.

To confirm these observations, the fluorescence intensities of the recombinant virus infected Sf21 cell lysates were quantified by spectrofluorometry. As depicted in Figure 2G, significant levels of the fluorescence were found in the lysates of vCMV-DRhir(L3-4)E and vCMV-DRhir(L3-6)E infected Sf21 cells. Specifically, fluorescence intensities from the lysates of vCMV-DRhir(L3-4)E and vCMV-DRhir(L3-6)E infected Sf21 cells were about 63% and 83%, respectively, of that of the lysates of vAcCMV-DRhirE infected Sf21 cells. In contrast, the fluorescence intensities of the cell lysates of vCMV-DRhir(L5-6)E, vCMV-DRhir(L6)E as well as the vCMV-DRhir(L1-2)E infected Sf21 cells, were barely detectable (Figure 2G). Consistent with this observation, Western blot analysis also revealed that EGFP proteins were expressed in the vCMV-DRhir(L3-4)E and vCMV-DRhir(L3-6)E infected Sf21 cells but not in the vCMV-DRhir(L1-2)E, vCMV-DRhir(L5-6)E and vCMV-DRhir(L6)E infected Sf21 cells (Figure 2H). Taken together, the L3, L4 tandem repeat TAAG motifs, but not the other four TAAG motifs in the RhPV 5′ IRES cDNA, are responsible for the promoter activity in baculovirus-infected Sf21 cells. We therefore named this short 110 bp cryptic promoter (nt 309–418 of RhPV 5′ IRES cDNA) as the RP110 promoter.

**Figure 3:**
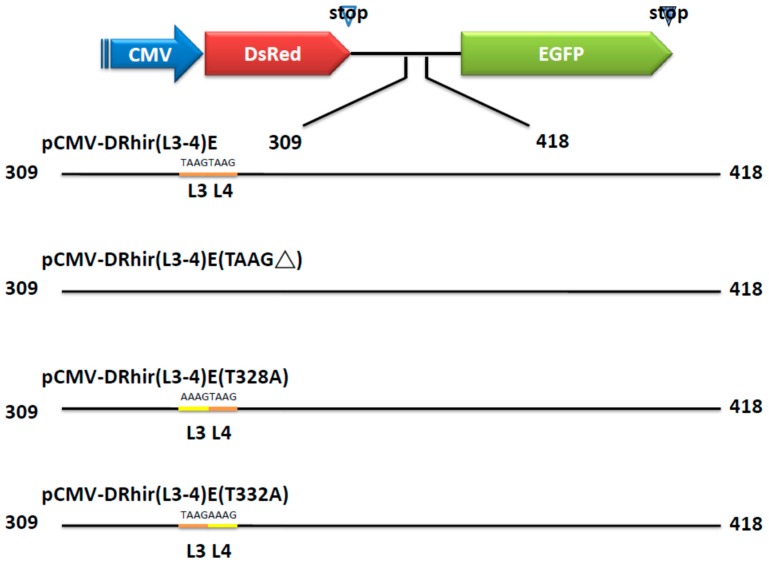
Schematic presentation of the truncations and mutations in the RP110 cryptic promoter. The indicated cDNA fragments were generated by chemical synthesis and inserted between the *DsRed* and *EGFP* genes in the bicistronic vector pCMV-DRhir(L3-4)E which containing the 309–418 bps of RhPV IRES cDNA fragment and the two tandem repeated TAAG motifs L3 and L4. pCMV-DRhir(L3-4)E (TAAG∆) indicated the L3 and L4 TAAG were deleted. pCMV-DRhir(L3-4)E (T328A) denoted the 328th nucleotide T was change to A and pCMV-DRhir(L3-4)E (T332A) denoted the 332th nucleotide T was changed to A.

### 2.2. TAAG Motif in L3 Is More Critical for RP110 Promoter Activity in the Baculoviruses Infected Sf21 Cells

Prior studies indicated that IRES elements derived from cellular mRNA, e.g., p27^kip1^ [21] and *pim-1* [22], possess cryptic promoter activity. Interestingly, a previous study confirmed that promoter activity is present in the cDNA sequence corresponding to the HCV IRES [23]. Our study provides another example of a cDNA sequence of an IRES element derived from RNA viruses acting as a promoter. A striking characteristic of a baculovirus late or very late promoter is its short length, e.g., the respective sizes of the polyhedron promoter and p10 promoter are about 89 and 114 bp. Thus, our finding of RP110 promoter is merely 110 bp in size is consistent with the size of a baculovirus late or very late promoter. Further, it is known that the transcription activity of the baculovirus late or very late promoter depends on baculovirus early gene expression. Thus, our finding that Sf21 cells transfected with pCMV-DRhirE or pCMV-DRhir(L3-4)E did not elicit red or green fluorescence (data not shown), suggests that the RP110 promoter acts as a baculovirus late or very late promoter, and its transcription activity depends on baculovirus infection. To further examine whether L3, L4 tandem repeat of TAAG is necessary for the RP110 promoter activity, the L3L4 deleted transfer vector pCMV-DRhir(L3-4)E(TAAG∆) was constructed (Figure 3); and used to generate the recombinant baculovirus vCMV-DRhir(L3-4)E (TAAG∆).

**Figure 4:**
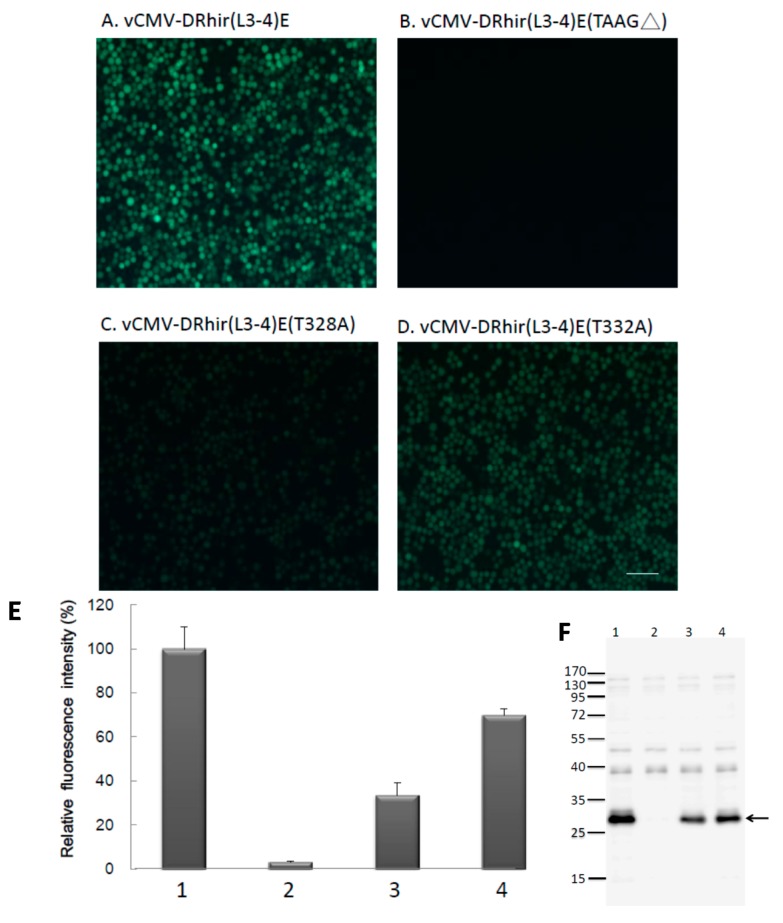
(**A**–**D**) The L3 TAAG motifs but not the L4 in the cDNA of RhPV 5′ IRES are dominant for RP110 promoter activity in baculovirus infected Sf21 cells. Fluorescence micrographs of Sf21 cells infected with (**A**) vCMV-DRhir(L3-4)E; (**B**) vCMV-DRhir(L3-4)E(TAAG∆); (**C**) vCMV-DRhir(L3-4)E(T328A); and (**D**) vCMV-DRhir(L3-4)E (T332A) at a multiplicity of infection of 1 at 3 days post-infection. Pictures were taken under a FITC channel with a 450/490-nm filter set using an exposure time of 900 ms; scale bar is 50 μm; (**E**,**F**) Quantitative analysis of promoter activity in the truncated and mutations in the RP110 cryptic promoter by spectrofluorometer and Western blot; (**E**) Spectrofluorometric measurement of EGFP fluorescence in lysates (100 μL; 50 μg of protein) from Sf21 cells infected with (1) vCMV-DRhir(L3-4)E, (2) vCMV-DRhir(L3-4)E (TAAG∆), (3) vCMV-DRhir(L3-4)E(T328A), or (4) vCMV-DRhir(L3-4)E (T332A) at a multiplicity of infection of 1 at 3 days post-infection. EGFP fluorescence emission was excited at 488 nm and monitored at 507 nm. The fluorescence intensity of EGFP was normalized to that of vCMV-DRhir(L3-4)E infected Sf21 cell lysates (100%); (**F**) Western blot analysis of cellular lysates from Sf21 cells infected with vCMV-DRhir(L3-4)E (Lane 1), vCMV-DRhir(L3-4)E (TAAG∆) (Lane 2), vCMV-DRhir(L3-4)E (T328A) (Lane 3), vCMV-DRhir(L3-4)E(T332A) (Lane 4). Proteins (25 μg per lane) were separated by 12% SDS–PAGE and then electrotransferred onto PVDF membranes, which were then incubated with rabbit anti-GFP polyclonal antibody followed by an HRP-conjugated goat anti-rabbit secondary antibody. Detected protein bands were visualized on X-ray film using a Western blot chemiluminescence kit. Molecular mass markers (kDa) are indicated at left and the arrow indicated the EGFP protein band.

The vCMV-DRhir(L3-4)E (TAAG∆) infected Sf21 cells did not emit any green fluorescence (Figure 4B), which is contrast to the vCMV-DRhir309E infected Sf21 cells (Figure 4A). This result indicated the L3, L4 tandem repeat TAAG motifs in the cDNA of RhPV IRES is necessary and sufficient enough to be a cryptic promoter in baculovirus infected Sf21 cells. Further investigation was directed to differentiate which of the L3 and L4 TAAG motifs is more important for the cryptic promoter activity. In this regard, two plasmids, in which a single mutation in either L3 or L4 were constructed without changing the size of RP110 promoter. Specifically, in pCMV-DRhir(L3-4)E (T328A), T at position 328 was mutated to A in L3; whereas in pCMV-DRhir(L3-4)E(T332A), T at position 332 was mutated to A in L4 (Figure 3). The corresponding recombinant viruses generated therefrom were named vCMV-DRhir(L3-4)E(T328A) and vCMV-DRhir(L3-4)E(T332A), respectively. Interestingly, both vCMV-DRhir(L3-4)E(T328A) and vCMV-DRhir(L3-4)E(T332A) infected Sf21 cells emitted the green fluorescence (Figure 4C,D).

However, the fluorescence emitted from vCMV-DRhir(L3-4)E(T328A) infected cells is weaker than that by vCMV-DRhir(L3-4)E(T332A) or vCMV-DRhir(L3-4)E infected Sf21 cells. Spectrofluorometer quantization indicated the about 70% reduction in fluorescence intensity found in vCMV-DRhir(L3-4)E(T328A) infected cells compared to that in vCMV-DRhir(L3-4)E infected cells. However, only about a 30% reduction in the fluorescence intensity was found in vCMV-DRhir(L3-4)E(T332A) infected Sf21 cells (Figure 4E). Similar results were found via probing the EGFP protein expression in the vCMV-DRhir(L3-4)E, vCMV-DRhir(L3-4)E(TAAG∆), vCMV-DRhir(L3-4)E(T328A) or vCMV-DRhir(L3-4)E(T332A) infected Sf21 cells with Western Blot analysis (Figure 4F). Thus, the L3 TAAG motif was found to be more critical for the promoter activity in the baculoviruses infected Sf21 cells.

### 2.3. RP110 Acts as an IRES in CHO Cells

**Figure 5:**
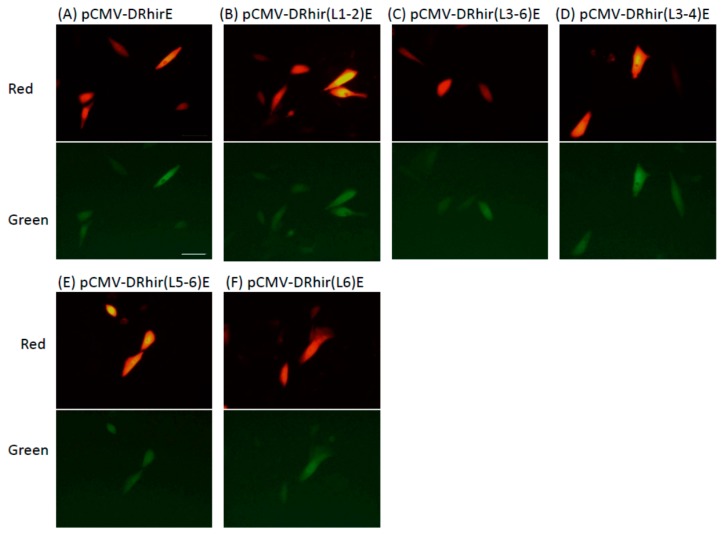
The truncated RhPV 5′UTR IRES element fragments displayed internal translational activity in mammalian cells. CHO cells (5 × 10^4^ cells per well, seeded in a 24-well plate) were transfected with pCMV-DRhirE (**A**); pCMV-DRhir(L1-2)E (**B**); pCMV-DRhir(L3-6)E (**C**); pCMV-DRhir(L3-4)E (**D**); pCMV-DRhir(L5-6)E (**E**) or pCMV-DRhir(L6)E (**F**) and observed at two days after transfection under fluorescence microscopy. Pictures were taken in the same field using a conventional rhodamine channel (Red) with a 510/560-nm filter set and a FITC channel (Green) with a 450/490-nm filter set. All pictures were taken with the same exposure time (900 and 400 ms for EGFP and DsRed, respectively). Scale bar is 50 μm.

A previous study indicated that a small fragment in the RhPV 5′UTR IRES, corresponding to nts 425–579, retains significant IRES activity in mammalian, plant and insect translation systems [24]. Furthermore, Groppelli *et al.* demonstrated that a region within the RhPV 5′UTR IRES can direct internal translation initiation [24]. The M300/429 fragment that spans the RP110 promoter region also exhibited IRES activity in all of the tested translation systems [24]. Our previous studies also demonstrated that the 579 nts RhPV 5′UTR function as an IRES, but not as a promoter, in mammalian cells [19]. Thus, it would be interesting to explore whether the 110 nt RP110 promoter or any other constructs listed in Figure 1A can function as an IRES element in mammalian cells, given that no promoter activity was found in vCMV-DRhir(L1-2)E, vCMV-DRhir(L5-6)E or vCMV-DRhir(L6)E infected Sf21 cells (Figure 2). Transient transfection of CHO cells with any of the truncated constructs listed in Figure 1 emitted red fluorescence and green fluorescence (Figure 5A–F). These transient transfection results support previous findings obtained using a rabbit reticulocyte lysate (RRL) *in vitro* translation system, which indicated that a good part of the RhPV 5′UTR may be deleted without significantly affecting its IRES activity. Interestingly, the 110 nt RP110 promoter can mediate IRES activity in CHO cells (Figure 5D). This result implies that the novel baculovirus RP110 promoter identified in the RhPv 5′UTR cDNA can function as an IRES in mammalian cells and may thus facilitate the development of a “shuttle” bi-cistronic baculovirus gene expression or delivery vector.

Identification of this novel RP110 promoter in the RhPV 5′UTR cDNA will impact baculovirus biology basic research and applications using baculovirus-based gene expression and gene delivery vectors. Among the six TAAG motifs in the DNA sequence of RhPV 5′UTR IRES, only the L3, L4 tandem repeat TAAG possess promoter activity in baculovirus-infected Sf21 cells. It has been proposed that if TAAG sequences in an appropriate context are present within a gene, transcription can be initiated within that gene. Linker-scan mutational analyses indicated that the TAAG motif is absolutely essential for transcriptional initiation to occur and that it appears to be the primary determinant of transcriptional initiation from baculovirus late promoters [25]. For example, the vp39 late promoter has three transcriptional initiation sites, each starting within a TAAG site. The identified 110 bp fragment may serve as a model for the artificial design of enhanced baculovirus promoters. Furthermore, baculoviruses are not only used in recombinant protein production, but are also recognized as potential mammalian gene delivery vectors. Expression of a gene of interest in a baculoviruses-based gene delivery vector is generally controlled by insect-inactive mammalian promoters, which complicates isolation and quantification of the recombinant virus. Use of this short functional RP110 promoter to drive recombinant gene expression in insect cells will facilitate virus selection and titer determination. By virtue of its IRES activity in mammalian cells, the transfected cells can be easily identified by the evaluation of downstream selection markers.

## 3. Experimental Section

### 3.1. Cells

The *Spodoptera frugiperda* IPBL-Sf21 (Sf21) cell line was cultured in TNM-FH insect medium containing 8% heat-inactivated fetal bovine serum. CHO (Chinese hamster ovary cells) cells were grown in Dulbecco’s modified Eagle’s medium (DMEM; Sigma, St Louis, MO, USA) containing 10% fetal bovine serum.

### 3.2. Construction of Plasmids

The different deletion of the cDNA of the RhPV 5′UTR IRES were amplified from the pCMV-DRhirE by PCR using the primer pairs listed in Table 1. After PCR reactions, these DNA fragments were treated with the *Eco*RI- and *Bam*HI and cloned into the *Eco*RI- and *Bam*HI-digested pCMV-DRhirE to replace the cDNA of the RhPV 5′UTR IRES. These plasmids including different TAAG motifs were named as pCMV-DRhir(L1-2)E, pCMV-DRhir(L3-6)E, pCMV-DRhir(L3-4)E, pCMV-DRhir(L5-6)E and pCMV-DRhir(L6)E (Figure 1). To identify which of the tandem TAAG motifs (L3 and L4) was more important for the cryptic promoter activity of RhPV 5′UTR IRES, we construct pCMV-DRhir(L3-4)E (TAAG∆), pCMV-DRhir(L3-4)E (T328A) and pCMV-DRhir(L3-4)E (T332A) plasmids (Figure 4). The DNA fragments of Rhir309 (TAAG∆), Rhir309 (T328A) and Rhir (T332A) that replace the cDNA of the RhPV 5′UTR IRES in the pCMV-DRhirE were generated by chemical synthesis (MDBio.Inc., Taiwan) and cloned into pUC57 vector, named as pUC57-Rhir309 (TAAG∆), pUC57-Rhir309 (T328A) and pUC57-Rhir (T332A). Then these pUC57 vectors were digested with *Eco*RI and *Bam*HI and cloned into the *Eco*RI- and *Bam*HI-digested pCMV-DRhirE and got the plasmids listed in Figure 3.

### 3.3. Recombinant Virus Production and Titer Determination

Using Cellfectin (1 μL), the Sf21 cells (2 × 10^5^ cells/well in a 24-well plate) were cotransfected with the linearized viral DNA Bac-N-Blue (0.25 μg; Invitrogen, Carlsbad, CA, USA) and 0.8 μg of one of the transfer vectors, pCMV-DRhir(L1-2)E, pCMV-DRhir(L3-6)E pCMV-DRhir(L3-6)E, pCMV-DRhir(L3-4)E, pCMV-DRhir(L5-6)E, pCMV-DRhir(L6)E, pCMV-DRhir(L3-4)E(TAAG∆), pCMV-DRhir(L3-4)E (T328A) and pCMV-DRhir(L3-4)E(T332A). The resulting viruses were named vCMV-DRhir(L1-2)E, vCMV-DRhir(L3-6)E, vCMV-DRhir(L3-4)E, vCMV-DRhir(L5-6)E, vCMV-DRhir(L6), vCMV-DRhir(L3-4)E(TAAG∆), vCMV-DRhir(L3-4)E(T328A) and vCMV-DRhir(L3-4)E(T332A), respectively. For the Bac-N-Blue viral DNA containing the *LacZ* gene controlled by the ETL promoter (baculovirus early to late promoter), these recombinant viruses were identified by X-gal staining according to the manufacturer’s protocol. The recombinant viruses were selected and purified by a series of end-point dilutions. Sf21 monolayers were used for virus propagation, and all viral stocks were prepared and titers determined according to the end-point dilution as described before [26].

### 3.4. Western Blot Analysis

Proteins were separated by SDS–PAGE on a mini Protein III system (Bio-Rad, Hercules, CA, USA). After SDS–PAGE fractionation, proteins were electrotransferred onto a polyvinylidene difluoride (PVDF) membrane (Millipore, Bedford, MA, USA). The resulting membranes were blocked with Tris-buffered saline (TTBS; 100 mmol/L Tris (pH 7.4), 100 mmol/L NaCl, and 0.1% Tween 20) containing 5% (*v*/*v*) non-fat, dry milk at room temperature for 1 h with gentle shaking. Subsequently, the membrane was incubated with 1:3500-diluted anti-EGFP antibody (BD Biosciences, San Jose, CA, USA) for the cryptic promoter activity in Sf21 cells. The antibody was diluted in TBS with 0.5% (*v*/*v*) non-fat, dry milk and incubated shaking at 4 °C overnight. Unbound antibodies were removed by 3 washes each of 10 min in TTBS buffer at room temperature with shaking. Then the membrane was incubated with 1:2500-diluted horseradish peroxidase (HRP)-conjugated secondary antibodies (BD) for 1 h at room temperature. The HRP on the membrane was detected by an enhanced chemiluminescence kit (Pierce, Rockford, IL, USA) following the protocol provided by the manufacturer (Fusion-SOLO, Newberg, OR, USA).

### 3.5. Transfection of CHO-K1 Cells

Transfections of CHO-K1 cells were performed using Lipofectine reagent (Invitrogen). The cells (at 5 × 10^4^/well) were plated onto 24-well plates. Before transfection, the cells were repeatedly washed with serum free media to remove all traces of sera. 1 μg of plasmid was diluted in 100 μL serum-free DMEM medium, and then Lipofectine reagent (1 μL) was added. The DNA-Lipofectin mix was incubated for 30 min for DNA-Lipofectin complex formation. Then the DNA-Lipofectin complex solution was transferred to the cells at a total volume of 500 μL by adding serum-free medium. After 5 h, the medium was removed and 500 μL fresh medium with 10% fetal bovine serum was added. One day post-transfection, these transfected cells were observed under fluorescence microscopy (Nikon, Shinagawa, Tokyo, Japan).

### 3.6. EGFP Reporter Assay

For measurements of the EGFP expression, Sf21 cells (2 × 10^5^ cells) were grown in 24 well plates and infected with the tested recombinant viruses at a multiplicity of infection (MOI) of 1. These infected cells were washed with phosphate-buffered saline (PBS) and lysed with 100 μL CytoBuster buffer (Merck Millipore, Kenilworth, NJ, USA). The fluorescence intensities of EGFP preparations were quantified by Cary Eclipse Fluorescence spectrophotometer and protein concentration was determined by BCA protein assay kit (Thermo Fisher scientific, Waltham, MA, USA). All green fluorescence intensity were normalized with total protein (50 μL extract).

## 4. Conclusions

A fragment of 110 bp (nucleotides 309 to 418) was identified in the cDNA of RhPV 5′UTR sequence to be the shortest fragment responsible for the late promoter activity in baculovirus infected Sf21 cells. This 110 bp fragment contains a TAAG tandem repeat that retains more than 60% of the late promoter activity of the full length RhPV 5′UTR sequence. IRES activity also remained unchanged in all truncated RhPV 5′UTR constructs. The observation that the novel baculovirus promoter identified in the RhPV 5′UTR can also function as an IRES in mammalian cells may facilitate the development of a “shuttle” bi-cistronic baculovirus gene expression or delivery vector.
